# Interoception: Where do we go from here?

**DOI:** 10.1177/17470218231172725

**Published:** 2023-05-09

**Authors:** Jennifer Murphy

**Affiliations:** Department of Psychology, Royal Holloway, University of London, Egham, UK

**Keywords:** Interoception, interoceptive accuracy, interoceptive awareness, body perception

## Abstract

In recent years, there has been a significant rise in interest in interoception, the processing of internal bodily signals. This interest has been coupled by increased concerns regarding the measurement and conceptualisation of interoception. Focusing on cardiac interoceptive accuracy, I outline what I believe to be the most pressing issues in the field of interoception—specifically the continued reliance on the heartbeat counting task. I then provide an overview of what I believe to be more general limitations concerning how we measure and conceptualise individual differences in interoception and suggestions for a way forward. Specifically, I believe that by moving beyond single measurements, establishing optimal levels of interoceptive accuracy, and refocusing from accuracy to propensity, we may be able to uncover the real-life relevance of interoceptive abilities.

In recent years, there has been exponential rise in interest in interoception, the processing of internal bodily signals (Brewer et al., 2021). Such interest is arguably driven by theoretical and empirical work that suggests interoception may be fundamental for various aspects of higher -order cognition (emotion, learning, and decision-making), with atypicalities in nteroception implicated in the aetiology of several physical and mental health conditions (e.g., Brewer et al., 2021; Critchley & Garfinkel, 2017; Murphy et al., 2017). Despite such interest, issues with the measurement and conceptualisation of interoception have hampered progress, with questions raised over the validity of commonly used measures of interoception (e.g., Brewer et al., 2021; Brener & Ring, 2016; Desmedt et al., 2018; Desmedt, Heeren, et al., 2022; Desmedt, Van Den Houte, et al., 2022; Gabriele et al., 2022; Ring & Brener, 1996). This paper provides an overview of what I consider to be the most pressing issues in the field and suggestions for what I believe to be the crucial next steps.

## Reliance on poor measures

Most models of interoception separate interoceptive accuracy (performance on behavioural tests of interoception) from self-reported interoception (questionnaire measures aimed at assessing participants’ beliefs regarding their interoceptive ability and engagement by interoceptive signals; e.g., Desmedt et al., 2022; Garfinkel et al., 2015; Murphy, Catmur, & Bird, 2019; Suksasilp & Garfinkel, 2022). While various other facets of interoception have been proposed (see Desmedt, Luminet, et al., 2022; Murphy, Catmur, & Bird, 2019; Suksasilp & Garfinkel, 2022), this paper focuses predominately on interoceptive accuracy—specifically cardiac interoceptive accuracy—as it has received the most research attention and remains dominant in the field.

Cardiac interoceptive accuracy is predominantly assessed using two main task formats: (1) tasks that ostensibly assess the perception of heartbeat sensations (e.g., counting or tapping procedures; Dale & Anderson, 1978; McFarland, 1975; Schandry, 1981) and (2) tasks that assess multisensory integration of heartbeat sensations and external cues (detection or discrimination procedures; hereafter for brevity ‘heartbeat detection tasks’; Brener et al., 1993; Brener & Kluvitse, 1988; Clemens, 1984; Whitehead et al., 1977; Yates et al., 1985). In the former, participants are asked to count the number of heartbeats that have occurred over a preceding interval. This is then compared to the number of objectively recorded heartbeats to determine accuracy. While there are multiple variants of the latter, typically heartbeat detection tasks require participants to determine whether an auditory or visual cue is presented in or out of sync with their heartbeat. Some heartbeat detection variants predefine intervals as synchronous and asynchronous to which responses are compared (e.g., 2 alternative force choice procedures (2AFC); Whitehead et al., 1977), and others present multiple intervals where the consistency of the interval selected (where it is above chance) is taken as a measure of accuracy (e.g., Brener et al., 1993).

Overwhelmingly, however, the heartbeat counting task continues to dominate the field. This is despite 30 years of evidence challenging the validity of this task as a measure of cardiac interoceptive accuracy. The issues with the heartbeat counting task have been well articulated across multiple papers published across the past 20 years (e.g., Desmedt et al., 2018; Desmedt, Van Den Houte, et al., 2022; Murphy, Brewer, et al., 2018; Ring & Brener, 1996; Ring et al., 2015; Windmann et al., 1999), but they surmount to the concern that this measure is greatly influenced by participants’ beliefs regarding expected heartbeat sensations and that heartbeat counting scores do not allow us to distinguish individuals who can perceive heartbeat sensations from those who achieve good performance via guessing or estimation strategies.

Proponents of this task often point to evidence that they argue supports the criterion validity, construct validity, and reliability of the heartbeat counting task (e.g., Ainley et al., 2020; Schulz et al., 2021). While there are too many papers to cite that defend the use of the heartbeat counting task, often authors draw on evidence of associations between the heartbeat counting task and detection procedures, insula activity, the heartbeat evoked potential, and “theoretically meaningful” variables, or point to evidence of reduced performance following disruptive transcranial magnetic stimulation (TMS) and evidence of reasonable test–retest reliability. Below I outline why I remain unconvinced by this evidence.

First, there is little evidence of a relationship between heartbeat counting and detection procedures. Indeed, a recent meta-analysis by myself and colleagues suggests only a small relationship between measures (*r* = .21; with no relationship observed when multi-level designs are employed; Hickman et al., 2020). One suggestion is that this small relationship reflects the different task demands, while the heartbeat counting task is presumed to assess perception of internal sensations, detection tasks require participants to perceive the internal sensation and then integrate it with an external stimulus. As such, heartbeat detection tasks are often presumed to be more difficult than the heartbeat counting task (Murphy et al., 2017; Suksasilp & Garfinkel, 2022). What follows from this argument is that individuals who are impaired on the heartbeat counting task (a presumed measure of *perception*) should be impaired on heartbeat detection task (a measure of *perception* and internal/external integration). Likewise, if a relationship or group difference is observed on the heartbeat counting task (a presumed measure of *perception*), a relationship or group difference should be observed when the heartbeat detection task is employed in the same sample (with this driven by the fact that both tasks assess heartbeat *perception*). However, evidence is not consistent with either assumption. Despite few studies employing multiple tasks (see Hickman et al., 2020), reports exist of individuals who have poor performance on the heartbeat counting task (<50% accuracy) and yet are classified as interoceptive on heartbeat detection procedures (Ring & Brener, 2018). Similarly, there are occasions where group differences are observed for the heartbeat counting task that are not replicated when heartbeat detection measures are employed (Hina & Aspell, 2019). This is somewhat surprising if both tasks are presumed to assess *perception.* While one may argue that differences in sustained attention may underlie differences (as the heartbeat counting task involves continued attention to heartbeat sensations), where control tasks have been employed (e.g., time estimation) there is not strong evidence that these unexpected dissociations are driven by differences in sustained attention (Hina & Aspell, 2019).

Proponents of the heartbeat counting task also point to the measure’s test–retest reliability (for an overview see Ferentzi et al., 2018; Murphy, Cheesman, et al., 2019)—though notably this ranges between ∼.41 to .81 -, with little consideration of what may drive good stability where observed. Indeed, it is generally accepted that interoception involves both state and trait effects (e.g., Brewer et al., 2021; Schulz et al., 2013; Wittkamp et al., 2018), in which case the high stability of the heartbeat counting task may be somewhat surprising. Given evidence that beliefs strongly influence performance on this task (see Brener & Ring, 2016), it seems plausible that the stability of one’s beliefs regarding one’s own and the average resting heart rate are more likely to underlie such high stability where observed.

Proponents also point to the well-established relationship between the heartbeat counting task and theoretically meaningful variables. Yet, there are logical and empirical issues with this argument; logically, this is a prime example of circular logic—the heartbeat counting task is valid because it relates to theoretically meaningful variables, my theory is correct because I observed the expected pattern with the heartbeat counting task. Even if one subscribes to such circular logic, evidence from large meta-analyses do not support such conjecture; when pooled, the existing evidence shows no relationship between the heartbeat counting task and theoretically relevant measures (Desmedt, Van Den Houte, et al., 2022), with the same true for the 2AFC heartbeat detection task and certain theoretically relevant outcomes (Adams et al., 2022).

Beyond behavioural evidence, evidence from neuroimaging or brain stimulation (TMS) is often drawn upon to support the validity of the heartbeat counting task (Coll et al., 2017, 2021; Pollatos et al., 2007). While reliance on such associations suffers from the general issue of reverse inference and circular logic, there are further issues with reliance on such data. Indeed, it is not possible to determine whether associations between the heartbeat counting task (or indeed other measures of interoceptive accuracy) and neural measures (insula activity; the heartbeat evoked potential [HEP]) are driven by attention to heartbeats (as attention to heartbeats also relates to the amplitude of the HEP; Coll et al., 2021) or accuracy specifically, and the extent to which associations may reflect cardiac dynamics—e.g., differences in physiology—that may influence both neural measures such as the HEP and cardiac interoceptive accuracy remains to be determined (for discussion see Coll et al., 2021). Most notably there is often selective reading of the literature; in the aforementioned meta-analysis, the only study that examined the relationship between HEP amplitudes (grand averages across conditions) and both heartbeat counting and detection tasks observed no relationship with either measure (Schulz et al., 2015). Such results are surprising when considering the pooled results and while there may be legitimate reasons for differential results across tasks (see Schulz et al., 2015), I have yet to come across any authors arguing that the heartbeat detection task represents an invalid measure of interoception due to its lack of relationship with the HEP. While evidence that TMS disrupts heartbeat counting (and reduces the HEP amplitude) may be ostensibly seen as the best evidence for validity (Pollatos et al., 2016), in the absence of a control task (coupled with questions regarding the areas reached by stimulation), it remains questionable whether such effects are specific to interoception or instead reflect disruption to other brain areas that may subserve non-interoceptive processes that relate to performance on the heartbeat counting task (Coll et al., 2017).

Even if one ignores the aforementioned limitations of the evidence drawn upon to support the continued use of the heartbeat counting task, the issue remains that it is impossible to determine whether a high score on the heartbeat counting reflects better interoceptive accuracy or good performance achieved via non-interoceptive means; while it is arguably less likely that an interoceptive participant will score poorly on the heartbeat counting task (though as noted above, it does surprisingly seem to occur in some cases; Ring & Brener, 2018), a completely unknown proportion of individuals who score well can perceive heartbeats. While genuine associations may emerge where the proportion of “truly” interoceptive participants exceeds the noise created by the variable processes that could contribute towards good performance (and this may explain some of the aforementioned evidence supporting the task), this unknown proportion will likely vary across studies. This variability could feasibly contribute towards false associations (where general processes like intelligence—that relates to more accurate beliefs about typical heartbeats and better performance on the heartbeat counting task—relate to the comparison variable of interest; Murphy, Millgate, et al., 2018) and mixed results that are not possible to interpret. Although some may argue that cut-off scores could be used to determine a threshold at which we might have greater confidence that classifications do not reflect false positives (e.g., 85%–90% accuracy), empirical evidence supporting increased sensitivity using such methods has yet to be produced. Overall, the heartbeat counting task does not allow us to determine if an individual can perceive discrete heartbeats and thus tells us nothing about cardiac interoceptive accuracy.

It is my opinion that the field of interoception can do better than the heartbeat counting task, and I regret having relied solely on this task in much of my early work even when using several control measures (e.g., Murphy, Brewer, et al., 2018; Murphy, Cheesman, et al., 2019; Murphy et al., 2020). While some may argue that the task simply lacks specificity and there may be situations where there is a preference for a false positive (deeming someone interoceptive who is not) than a false negative, reliance on this measure has created a replication crisis in the field of interoception—one simply cannot trust results obtained using this task and one cannot easily interpret results where an individual or group has difficulties with the heartbeat counting task and not the heartbeat detection task even where control tasks are employed. This is not to say that this is the only measure of interoception that has been criticised. Indeed, the 2-alternative forced choice version of the heartbeat detection task has been criticised for not considering individual differences in the delay at which individuals perceive a stimulus as synchronous with their heartbeat (Brener & Ring, 2016), measures of self-reported interoception have issues with individual differences in interpretation of items (Gabriele et al., 2022) and show little correspondence with each other (Desmedt, Heeren, et al., 2022), measures of interoceptive insight (confidence-accuracy relationships; Garfinkel et al., 2015) have been criticised for reliance on too few trials, and neural measures such as the heartbeat evoked potential have been criticised for a lack of consistency in pre-processing and analytical choices (Coll et al., 2021). More recent criticisms have gone further, highlighting the predominant issue that few (if any) tasks can decouple the size of the stimulus from the perception of the stimulus, rendering individual differences in internal “perception” muddied by differences in the strength of afferent signalling (Desmedt, Luminet, et al., 2022). While I would argue that the lastmentioned concern is perhaps overstated—individuals who have difficulties with perception or weak signals are likely to miss interoceptive information in their everyday lives regardless of the cause(s) of their difficulties—I would agree that interventions would need adapting and as such decoupling of perception and stimulus strength is ideal. Overall, this demonstrates that there are serious issues with measurement in the field of interoception not limited to the heartbeat counting task, but the heartbeat counting task remains the measure most commonly employed. This is likely due to its ease of administration, that many (myself included) have invested time collecting data using this measure, and that individuals may lack resources or time to focus on measurement—exciting theoretical questions are far easier to sell to funding committees. One can empathise with these reasons, but as interoception research gains prominence (Khalsa & Lapidus, 2016) and moves towards interventions (Quadt et al., 2021) there is a need for a more vigorous approach to re-evaluate the knowledge base on which such work is based. Work should be published that advances the field, and my remaining heartbeat counting data will remain where I believe it should—in the file drawer.

## Poor conceptualisation and measurement of individual differences

Issues with the measurement of interoception have ignited a renewed focus on the development of novel measures. Indeed, in the past few years, a great number of novel measures (not limited to cardiac interoception) or novel analytic approaches for existing tasks have been developed (e.g., Garfinkel et al., 2016; Khalsa et al., 2009; Legrand et al., 2022; Plans et al., 2021; Smith et al., 2020, 2021; Van Den Houte et al., 2021; for a review of these novel measures please see Desmedt et al., under review). Such work is exciting, with cross-domain work in various formats opening up the possibility of examining the unitary nature of interoceptive ability (potentially overcoming the issue of differing tasks demands that has thus far precluded strong conclusions about dissociations or unity) and extending work beyond the cardiac domain. It should be noted that this proliferation of measures has not necessarily improved interoception measurement as such measures still require careful evaluation before they can be embedded in research. By which criteria, they should be evaluated remains questionable (arguably we should be cautious of putting much weight on criteria such as test–retest reliability when empirical and theoretical work suggests the possibility of state effects; see Brewer et al., 2021), but I would argue that careful evaluation of the task design (determining if this appears to assess the facet of interest, the design controls for confounds etc.) and the presence of a control task (that at least controls for attentional/motivational effects if not matched for difficulty) are reasonable baseline criteria. However, beyond the measurement of interoceptive accuracy specifically, I believe there are outstanding issues in how we measure and conceptualise individual differences in interoception that apply to all measures. This includes the use of single measurements, a lack of clarity regarding what is the optimal level of interoceptive abilities, and neglect of the interaction between interoception and exteroception. It is these limitations that I focus on below.

When examining the question of whether individual differences in interoceptive accuracy relate to a psychological construct, interoceptive accuracy is typically assessed via a task on a single occasion that is then compared “offline” to the psychological construct of interest. Such an approach assumes that this single measurement provides an adequate assessment of trait-level differences in interoception. However, existing evidence from classic tasks attests that tasks are influenced by both trait and state-level factors (Brewer et al., 2021; Wittkamp et al., 2018) and far more assessments are needed to examine trait level effects. While one approach may be to aggregate scores, it is notable that ~70% of individuals do not pass the “gold standard” cardiac interoceptive accuracy tasks—being interoceptive is not the norm (Brener & Ring, 2016). Instead, as colleagues and I have proposed previously (Plans et al., 2021), it may be more useful to examine the number of situations where an individual is able to perceive interoceptive signals. An individual who does not perceive their heartbeat after significant exertion or a strong perturbation of the body’s internal state (for an example method see Khalsa et al., 2009) is far more likely to suffer from difficulties associated with poor interoeceptive ability. The current approach, that might suggest ~70% of people require interoceptive intervention, is not likely to be useful clinically.

When considering interventions, there remain questions regarding what is the optimal level of interoceptive ability. While it may be easy to describe simply as ‘the ability to perceive a change in the body’s internal state and respond accordingly’, it is arguable whether the current approach is able to capture or train this. There are multiple reasons why a move towards interoceptive training is beneficial for the field. Indeed, evidence from interoceptive training provides crucial causal evidence for the role of interoception in health (e.g., Quadt et al., 2021). While there is a need to examine the mechanism(s) by which training may improve factors such as anxiety, as interventions typically do not solely involve accuracy feedback and may target facets beyond accuracy such as interpretation or attention to signals (for discussion see Adams et al., 2022), such an approach importantly provides the opportunity to move beyond correlational evidence. Yet, as we focus on interoceptive training considering the multiple facets of interoception that exist (attention, accuracy, insight), it is important for the field to determine what the optimal level of interoception for these facets may be—more is not always better and there are ethical issues that require consideration. Indeed, evidence suggests that increased attention may be maladaptive (for discussion in relation to anxiety see Brewer et al., 2021) and one may envisage that heighted accuracy of perceiving internal bodily sensations, or overconfidence in one’s ability, is not likely to be useful in all situations. All interventions carry risks and benefits, but our knowledge of the optimal level of interoceptive abilities is limited, particularly where tasks are inadequate as discussed above. As well as reconceptualising what “poor” interoception is, and moving beyond single assessments, more work is required to determine what level is optimal, and who is likely to benefit, before interoceptive training can be considered a useful therapeutic.

Finally, it is worth considering that the above—where interoceptive accuracy is assessed via a task on a single occasion that is then compared “offline” to the psychological construct of interest—is likely to neglect individual differences in one’s propensity to use internal signals (Murphy, 2022). Indeed, typically when assessing interoceptive accuracy individuals are invited into the lab and explicitly asked to attend to internal signals. This tells us nothing about whether an individual uses these internal signals “online” in their everyday lives, nor whether they are likely relying on these signals when completing other task(s). Differences in propensity have been reported (most notably when considering gender differences; see Murphy, 2022; Pennebaker & Roberts, 1992; Prentice et al., 2022), and such individual differences are likely to provide far more useful insights regarding the relevance of interoception beyond the lab. After much focus on interoception, it is time to focus on the interaction of interoception and exteroception and individual differences in the ability to flexibility to attend to, and integrate, information from both streams.

In conclusion, it is my view that there are serious issues with the measurement of interoception. While these are not limited to the heartbeat counting task, the use of this measure continues to hamper progress. Methodological advances hold promise for future progress, but I would argue for a need to reconceptualise individual differences in interoception, moving beyond single measurements, establishing optimal levels and refocusing from accuracy to propensity to consider the interaction of interoception and exteroception and the real-life relevance of interoceptive abilities.
